# Salmagundi

**Published:** 1886-08

**Authors:** 


### SALMAGUNDI.

EDITED BY E. G. BETTY, D.D.S.

OXFORD UNIVERSITY, Eng., has also recently conferred honorary degrees upon Dr. Oliver Wendell Holmes.

EXPERIMENTS by Italian physiologists prove that cocaine destroys the sense of taste for bitter substances only.

THE Editor of the "Ohio State Journal of Dental Science" does more writing than any of his contemporaries because it is his "special" business.

DR. E. C. MOORE, of Detroit, a good friend of ours, paid Cincinnati a visit last month, and reports all things lovely in the beautiful Michigan metropolis.

DR. L. D. CAULK passed through Cincinnati on his return from Chicago, where he was in attendance upon the Dealer's Association, paying us a flying visit.

DR. WILL TAFT recommends a coarse burr dipped in olive oil as the best instrument for dressing down gold fillings; the olive oil preventing clogging of the burr better than any animal oil.

DR. W. D. KEMPTON is again afflicted. This time by the death of his venerable father, who departed this life on the night of the 29th of July. Dr. Kempton has the sympathy of hosts of friends.

MATHEW GIBSON, M.D., of Felicity, O., has been invited to London, Eng., to lecture at King's College. He is the first from Ohio, and one of the very few in the United States that have been thus honored.

The following play upon words from the "Lowell *Courier*" is good in other than a political sense. "Many a dentist can extract teeth without pain, but the Senate will find it cannot extract Payne without teeth."

---

The prospectus of "The American System of Dentistry, in Treatises by Various Authors," edited by W. F. Litch, M.D., D.D.S., is out, and judging by the list of topics embraced, bids fair to be a great credit to the profession.

---

We are now in the midst of the dog days, a time when one does not feel inclined either to eat, drink, sleep, or fill teeth, and so seeks rest and change at one of the many summer resorts. One of the very best summer resorts is that of the pen wielded in the cause of progress.

---

Are you aware that your patrons are enquiring why you do not attend the conventions? Not only that, they are beginning to think that you are ashamed to go, and they are becoming ashamed of you. You can't fool them much longer.—*Editorial in Southern Dental Journal.*

---

Physicians are now using aniline-oil as a local anaesthetic when simple operations, such as the opening of a felon, are to be performed. The finger, in such a case, is dipped for a short time in the oil, and, although the flesh may subsequently be cut to the bone, it is said there is absolutely no pain.—*Science.*

---

The improved absorbent cotton manufactured by Seabury & Johnson is a very valuable material to the dentist, as it supplies the need of an absorbent, a vehicle for medicaments, and acts admirably as a wedge for separating teeth. This may not be news to many, but there are others possibly who have not heretofore had the advantage of its use, and to them we heartily recommend it.

CORKS may be made ether-tight by coating them with a solution prepared from 4 parts of gelatin, 52 parts of boiling water, and 1 part of ammonium bichromate (added to the filtered gelatin solution) and then exposing them for a few days to sun light. Prepared in this way, corks will prevent the evaporation of any of the volatile fluids.

---

The following has considerable truth in it:

"THE foreign population have worse teeth than the native Americans," said a dentist on Third avenue, who was doing a land office business, to a Mail and Express reporter. "Why is it? Simply because the foreigners do not take any care of their teeth, never have them filled, and consequently lose them by decay. Americans, on the other hand, watch the teeth of their children, have them cared for early, and save a false set when they arrive at maturity. The Germans have bad teeth, many of them wearing false sets. Parents, as a rule, are to blame for their children's bad teeth. They neglect to have any work done until it is too late. The prevalence of false teeth is increasing to an alarming extent, and simply from negligence."

---

ENGLISH dentistry receives the following notice from *Science*, and we wish it were the same in this country:

Applicants for admission to the dental schools of Great Britain must pass a satisfactory examination in English grammar and history, in Latin, in algebra, geometry, and physics; and, before they can receive their degree of L.D.S., they must study for four years anatomy, chemistry, surgery, and such other branches as are taught in the medical schools, besides those which specially pertain to dentistry, as operative dentistry, the administration of anaesthetics. In London there are two dental hospitals in which all the operations known to that branch are practised, and to which students have admission and opportunity to operate. In the National dental hospital, during the year 1885, 9,001 fillings were inserted, of which 1,014 were of gold, the others being of gutta-percha or other plastic material.

Dr. Jennings, of Cleveland, not only fills the roots of dead and pulpless teeth with a solution of gutta-percha, but also fills the cavity formed in the alveolus by an abscess. This he does by a process of pumping, using as a piston, a broach bound with cotton. After thoroughly removing all the *debris* of a putrescent pulp, he washes out both cavity and canal with alcohol, a weak solution of carbolic acid or both, and then forces in the gutta-percha solution until it makes its appearance externally, either at the margin of the gum or fistulous opening as the case may be. His theory is that by filling with a benign and inert substance all space around the apex, there will be no possibility of an exudation that in turn will degenerate into pus. The idea is a novel one and worthy of a trial in many of those cases that give us no end of trouble, and frequently mortify us by our unsuccessful attempts to produce or rather induce a cure.

---

### STATES.

A gentle Miss, once seized with chill,
Was feeling very, very Ill.,
When came an Md. for to know
If N. Y. service he could do.

" O.," cried the maid (for scared was she),
" Do you Ind. Tenn. to murder Me.?"

" La." cried the doctor, " I Kan. save
You from a most untimely grave
If you will let me Conn. your case,
And hang this liver pad in place."

" Am Ia. fool?" the patient cried.
"I can not Del." the man replied;
" But no one can be long time Ill.
Who Tex. a patent blue Mass. pill."

" Ark.!" shrieked the girl " I'll hear no Mo.,
Your nostrums are N. J.--No go."

